# Systemic Inflammation Associates With a Myeloid Inflamed Tumor Microenvironment in Primary Resected Colon Cancer—May Cold Tumors Simply Be Too Hot?

**DOI:** 10.3389/fimmu.2021.716342

**Published:** 2021-08-31

**Authors:** Anne Helene Køstner, Patricia Switten Nielsen, Jeanette Baehr Georgsen, Erik Thorlund Parner, Mette Bak Nielsen, Christian Kersten, Torben Steiniche

**Affiliations:** ^1^Department of Oncology, Sorlandet Hospital, Kristiansand, Norway; ^2^Department of Pathology, Aarhus University Hospital, Aarhus, Denmark; ^3^Section for Biostatistics, Department of Public Health, Aarhus University, Aarhus, Denmark; ^4^Department of Oncology, Akershus University Hospital, Nordbyhagen, Norway

**Keywords:** systemic inflammation, C-reactive protein, multiplex, immunohistochemistry, colon cancer, myeloid inflammation, neutrophils, spatial profiling

## Abstract

Systemic inflammation measured by the acute-phase protein CRP associates with poor outcome across cancer types. In contrast, local tumor-associated inflammation, primarily evaluated by T-lymphocytes, correlates with favorable prognosis. Yet, little is known whether these two responses are related or opposing processes and why elevated CRP in relation to cancer is detrimental for clinical outcome. As proof of concept, we developed a platform combining multiplexed IHC and digital imaging, enabling a virtual readout of both lymphoid and myeloid immune markers and their spatial patterns in the primary tumors of resected stage II and III colon cancer (CC) patients with and without accompanying systemic inflammation. Twenty-one patients with elevated CRP (>30 mg/l) and 15 patients with low CRP (<10 mg/l) were included in the analyses. Whole slides from the primary tumors were stained for markers of adaptive (CD8+, CD4+, foxp3 regulatory T cells, CD20+ B cells) and innate (CD68+ macrophages, CD66b+ neutrophils) immunity and the immune checkpoint molecule PD-L1. Associations between individual immune markers, preoperative CRP values, mismatch repair status (MMR), and risk of recurrence or death were assessed. Unsupervised hierarchical clustering was used to explore whether distinct immune phenotypes were present. Tumors from systemically inflamed patients (CRP >30 mg/l) displayed significantly more myeloid features in terms of higher densities of CD66b+neutrophils (p = 0.001) and CD68+macrophages (p = 0.04) and less lymphoid features (lower CD8 T cell, p = 0.03, and foxp3 regulatory T cell densities, p = 0.03) regardless of MMR status. Additionally, systemically inflamed patients harbored lower mean distances between neutrophils and tumor cells within the TME. Intriguingly, microsatellite instable (MSI) tumor status correlated with systemic inflammation. However, using a combinatorial approach, we found that regardless of an adaptive composite score (compounded CD4+ and CD8+ T cells), a high innate score (CD66b+ neutrophils and CD68+ macrophages) associated significantly with elevated CRP. In conclusion, tumor-associated systemic inflammation correlated with a myeloid-dominated TME in a small cohort of resectable CC patients. Our data highlight the importance of a comprehensive immune classification of tumors including players of innate immunity and support a role for CRP as an informative biomarker of the immune response taking place at the tumor site.

## Introduction

The crucial role of the immune system in tumor biology and clinical outcome across cancer types is by now well accepted (1). Tumor-associated inflammation has traditionally been referred to as either a systemic inflammatory response (SIR) or a localized *in-situ* immune infiltrate. SIR, as evidenced by circulating biomarkers such as the acute-phase protein C-reactive protein (CRP), has consistently been correlated with poor prognosis in many cancer types, including colon cancer (2–4). In contrast, a robust intra-tumoral lymphocyte infiltrate associates with favorable prognosis and seems predictive of response to both chemotherapy and immune checkpoint blockade (5, 6).

In colon cancer, the prognostic significance of tumor-infiltrating T-lymphocytes has been extensively validated by Immunoscore, which has shown prognostic superiority to the classical TNM staging (7–9). Based on this scoring system, the concept of “hot” (T-cell inflamed) and “cold” (no/little tumor infiltrating T-cells) tumors has emerged with accumulating studies using this T-cell-focused model for categorizing the immune landscape and predicting treatment outcome in a wide range of cancer types (10).

However, the immune infiltrate of most solid tumors is highly heterogeneous and dynamic (11). Apart from T-cells and other adaptive immune cells, it consists of innate immune cells such as neutrophils, macrophages, and dendritic cells, which together with fibroblasts, endothelial cells, and other stromal components constitute the complex tumor microenvironment (TME) (11, 12). Myeloid immune cells in particular exhibit remarkable plasticity with the ability to polarize into functionally distinct phenotypes either supporting or inhibiting tumor growth depending on the signals in the TME (13). Despite their possible dual roles in cancer development, most studies point toward a dominating tumor-promoting and immunosuppressive role of myeloid immune cells in the TME (13, 14).

Nevertheless, in the era of immune checkpoint blockade where preexisting T-cell-mediated immunity is key for therapeutic efficacy, the impact of innate immune cells on tumor progression and treatment outcome has been less appreciated. Furthermore, adding another layer of complexity, recent studies have highlighted the importance of characterizing the spatial distribution of immune cells within the tumor, to understand how tissue architecture and cellular interactions may shape the immune landscape (15, 16).

Given this diversity of the tumor-immune microenvironment in terms of various immune cell populations, their spatial organization, and the dual role they may play in cancer, it is desirable to identify biomarkers and develop diagnostic tools that reflect the inherent immunological status of tumors. Specifically, indications of either a myeloid- or lymphoid-dominated microenvironment and their respective immune-suppressive or stimulatory capacities may prove to be the cornerstone for allocating patients to the most appropriate treatment strategies.

The aim of this study was therefore to explore the immune contexture as a whole, featuring both adaptive and innate players in the TME of primary resected colon cancer patients with and without associated SIR. For this purpose, we developed a multiplex immunohistochemistry (mIHC)-based platform combining chromogenic IHC staining with digital whole-slide imaging enabling simultaneous detection of six different lymphoid and myeloid immune cells in addition to the immune checkpoint molecule PD-L1. Using this platform, we were able to characterize the immune landscape and assess spatial relationships in the TME of the primary tumors. We further extended the application by combining the mIHC data with clinical information to investigate whether SIR and local tumor-associated inflammation are related processes and explore the hypothesis that SIR correlates with a myeloid-driven immune landscape in colon cancer patients.

## Materials and Methods

### Patients and Tumor Specimens

Forty-three stage II and III colon cancer patients, consisting of 20 patients with CRP < 10 and 23 patients with CRP > 30 treated at Sørlandet Hospital, Kristiansand, Norway, were selected from a prospective local colorectal cancer database covering extensive clinical information and follow-up data. The choice of CRP values was based on previous work using identical CRP thresholds (2). All patients had been resected for their primary tumors between 2005 and 2015 as an elective procedure and neither had received antibiotics nor immunosuppressive drugs within the last month prior to surgery nor had been diagnosed with an autoimmune disease. CRP values were obtained up to 20 days before the resection.

Archived formalin-fixed, paraffin-embedded (FFPE) tumor tissues from the primary tumors were retrieved from the Department of Pathology, Sørlandet Hospital. Representative tumor blocks containing areas of both the invasive margin (IM) and tumor center (TC) were selected by a trained pathologist (MBN).

The study was conducted according to approvals from the Regional Ethics Committee.

### Multiplex Immunohistochemistry Workflow

FFPE colon cancer blocks were cut into 3 mm thick sections and prepared for the IHC-staining protocol. All staining procedures were performed on the Ventana Discovery Ultra autostainer (Roche Diagnostics International AG, Switzerland).

First, tissue sections were deparaffinized using xylene and rehydrated with ethanol followed by heat-induced antigen retrieval and blocking endogenous peroxidase activity. Then, mIHC with two different panels of antibodies were applied on two serial tumor sections. The first panel consisted of a 5-plex termed the adaptive or lymphoid immune profile with primary antibodies against CD8 (cytotoxic T lymphocytes), CD4 (T-helper cells), foxp3 (regulatory T cells), CD20 (B lymphocytes), and pan-cytokeratin (pan-CK) as an epithelial tumor marker. The second IHC panel, a 4-plex termed the innate or myeloid immune profile, consisted of antibodies against CD68 (pan-macrophages), CD66b (neutrophils), pan-CK, and finally PD-L1. The multiplex staining process consisted of sequential staining rounds with primary and secondary antibodies (see **Table S1** for details), without hematoxylin counterstaining to prevent mix of signals in the digital analysis. After accomplishing the multiplex IHC procedure, tumor sections stained with the innate immune panel were counterstained with hematoxylin for visualization of nuclei and tissue architecture. Three forms of controls were used to assure the staining quality of the multiplex: 1) comparison with single staining for each of the markers to check for cross-reactivity or loss of signal due to the multiplex procedure, 2) applying tonsil tissue as a “positive control” on each slide (consists of lympho-epithelial structures with cells positive for all of the markers included in the multiplex panels), and 3) mIHC staining of tumor tissue from lung (adenocarcinoma) for assay validation and grading of PD-L1 expression.

### Digital Imaging and Automated Analysis of the Tumor Immune Microenvironment

After completion of the staining process, tumor sections were scanned as bright-field whole slides at ×20 magnification using a NanoZoomer 2.0 HT (Hamamatsu, Japan). Image analysis was performed using Visiopharm Integrator System software version 2019.02 (VIS; Visiopharm A/S, Hoersholm, Denmark).

**Figure 1:**
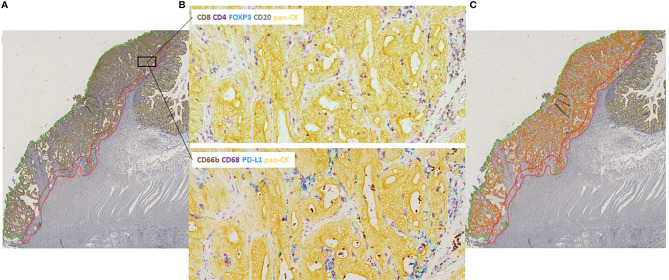
Tumor regions and image acquisition. **(A)** Hematoxylin-stained whole slide of stage II colon cancer with annotated invasive margin, IM (red), and tumor center, TC (green). **(B)** Two multiplexed IHC-stained serial slides from the same tumor area visualizing adaptive and innate immune markers. IHC-stained slides were digitally aligned together with the hematoxylin-counterstained innate slide. **(C)** Automated digital analysis was applied enabling one virtual readout of the IHC-stained markers. Immune cells were quantified at the IM and TC separately and classified as either directly intra-tumoral or embedded in the stoma.

As shown in **Figure 1**, the invasive margin (IM) and tumor center (TC) were manually outlined by an experienced pathologist (MBN) and the observer on hematoxylin-stained slides in the software. As for annotating the IM, we chose not to do that automatically using a predefined and fixed area measurement since the tumors showed considerable variability in size and range of tumor islets and stroma.

The two IHC-stained tumor sections and the hematoxylin-counterstained slide were scanned separately. Tumor slides were then digitally superimposed using an automated approach, but controlled and optimized manually, with the net effect of a single virtual slide capturing all seven immunostained markers with preserved tissue architecture.

Digital analysis was performed using applications within the software particularly developed for this material. For segmentation, we used a Bayesian classifier followed by different post-processing steps (primarily morphological operations and changes by area or surrounding) for optimizing the results. The preprocessed images in adaptive stains were based on color deconvolution of the chromogens DAB and silver in addition to features of the RGB color model. In innate stains, RGB and HSI models were utilized. The software-based classification of the immunostained markers was performed by assigning different pseudo-colors, enabling a visual output of the various immune markers within the tissue. Areas with mucin, artifacts, or tissue folds were manually excluded from the analysis.

Immune cell densities were estimated as area of positively stained cells per region of interest (ROI) in percent quantifying cells at the IM and TC separately. Immune cells were classified as either intra-tumoral (IT) if they were directly infiltrating the tumor nests or stromal (S) if they were located within the stromal spaces. In addition, we calculated two forms of a composite score: one with the sum of IT and S immune cells divided by the total area of the ROI of interest, and one where the area of tumor tissue was subtracted to adjust for differences in total amount of tumor tissue which potentially could dilute the true immune cell estimate. PD-L1 expression on tumor cells and immune cells (primarily CD68+ macrophages) was assessed separately. Composite lymphoid and myeloid immune scores were estimated by compounding the densities of CD8+ and CD4+ T cells for the lymphoid score and CD68+ macrophages (total score) and CD66b+ neutrophils for the myeloid score and categorized as high or low based upon the median value of the respective compounded scores.

Using pan-CK in the mIHC panels, distances between tumor- and immune cells of interest could be estimated enabling spatial characterizations. Two different types of spatial analysis were performed: 1) proximity analysis estimating the density of immune cells of interest within the defined distance of 20 micron around the tumor islets and 2) nearest neighbor analysis calculating the average distance between immune cells of interest and nearest tumor cell.

The tumor–stroma ratio was calculated by dividing the stromal area of the IM and TC by the total area of the two tumor compartments.

### Microsatellite Instability Analysis

Assessment of mismatch repair (MMR status) was performed by IHC evaluation of MHL1, MSH2, MSH6, and PMS2 protein expression. Tumors that were negative in one or more of the four stainings or inconsistent with IHC were verified with the Idylla MSI test, which is a fast-track PCR-based assay for determining microsatellite status in colorectal cancer (17).

### Statistical Analysis

Differences in clinicopathological data between CRP-high and -low patients were evaluated by Fisher’s exact test and the two-sample t-test. Immune markers were analyzed on the logarithmic scale to obtain a normal distribution. Associations between immune markers, CRP, and survival were analyzed by Fisher’s exact test. Pearson’s correlations were used to analyze the correlation between individual immune markers. Medians and means were compared using the Kruskal–Wallis test and the one-way ANOVA-test, respectively. The Aalen–Johansen method was used to estimate the risk of recurrence or death by colon cancer, adjusting for death of other causes as competing risk, and compared between CRP groups using the log-rank test. For estimating the lymphoid and myeloid composite scores, data were log-transformed and standardized before summing the score of the respective immune markers (CD8+/CD4+ T cells and CD68+ macrophages/CD66b+ neutrophils). To define subgroups in our cohort, unsupervised hierarchical clustering was performed. Heat maps and hierarchical clusters were generated in R studio version 4.0 based on the logarithmic scale of the immune markers standardized to mean zero and variance 1. Two-sided p-values < 0.05 were considered statistically significant for all analyses. Statistical analysis was performed using STATA software version 16.

## Results

**Figure 2:**
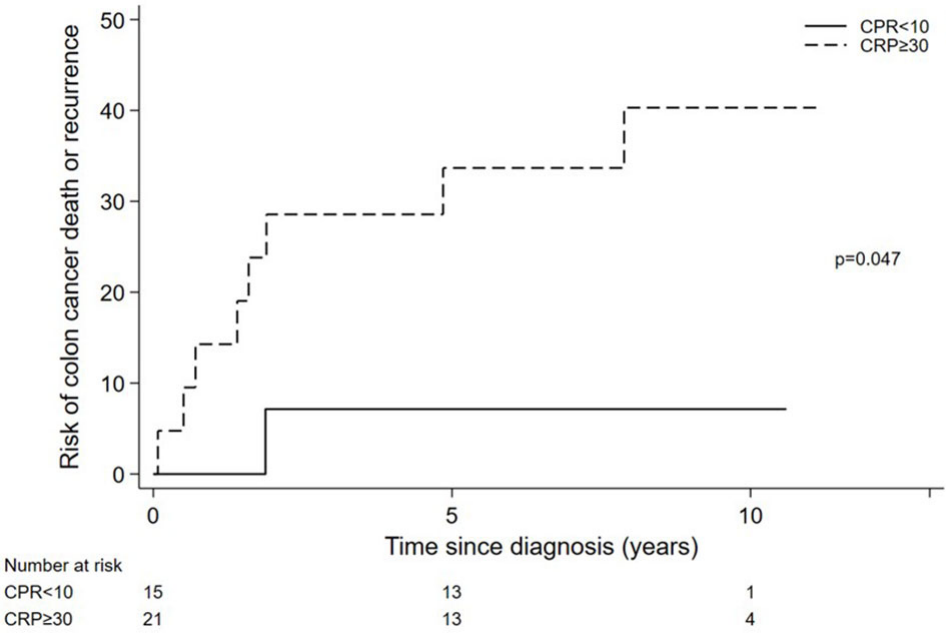
Risk of recurrence or death from colon cancer in CRP high and low patients.

A total of 36 stage II and III colon cancer patients were finally included in this study. Excluded patients (n= 7) were due to compromised tumor tissue quality, weak IHC staining, or other technical issues with the multiplex assays. Patient and tumor characteristics are listed in **Table 1**. Systemically inflamed patients were older and tended to be more right sided. Of note, all patients in the CRP-low group (n=15) had stage II disease while this was the case for only half of the patients in the CRP-high group (n=21). Nine of the patients in the systemically inflamed group had microsatellite instable (MSI-high) tumors, whereas all non-inflamed patients had microsatellite stable (MSS) tumors. As expected from previous works (2, 18), systemically inflamed patients had statistically increased risk of recurrence or death by colon cancer (see **Figure 2**, p=0.047).

**Table 1:** Patient and tumor characteristics in CRP high and -low colon cancer patients.

	CPR < 10	CRP ≥ 30	p value
	(n = 15)	(n = 21)	
**Age, mean (years)**	68	77	0.02
**Sex**			
Female	8 (53)	12 (57)	1.00
Male	7 (47)	9 (43)	
**Stage**			
II	0 (0)	10 (48)	0.002
III	15 (100)	11 (52)	
**Tumor location**			
Left	4 (27)	2 (10)	0.47
Right	8 (53)	13 (62)	
Sigmoid	3 (20)	6 (29)	
**Adjuvant chemotherapy**			
None	3 (20)	17 (81)	<0.001
Only 5-FU based	4 (27)	3 (14)	
Platinum doublet	8 (53)	1 (5)	
**Follow-up, mean (years)**	7.2	7.3	0.92
**MMR-status (MSS/MSI)**	15/0	12/9	<0.01

### Multiplex IHC Reveals Substantial Intra- and Intertumoral Heterogeneity of Immune Infiltration in Colon Cancer Patients

**Figure 3:**
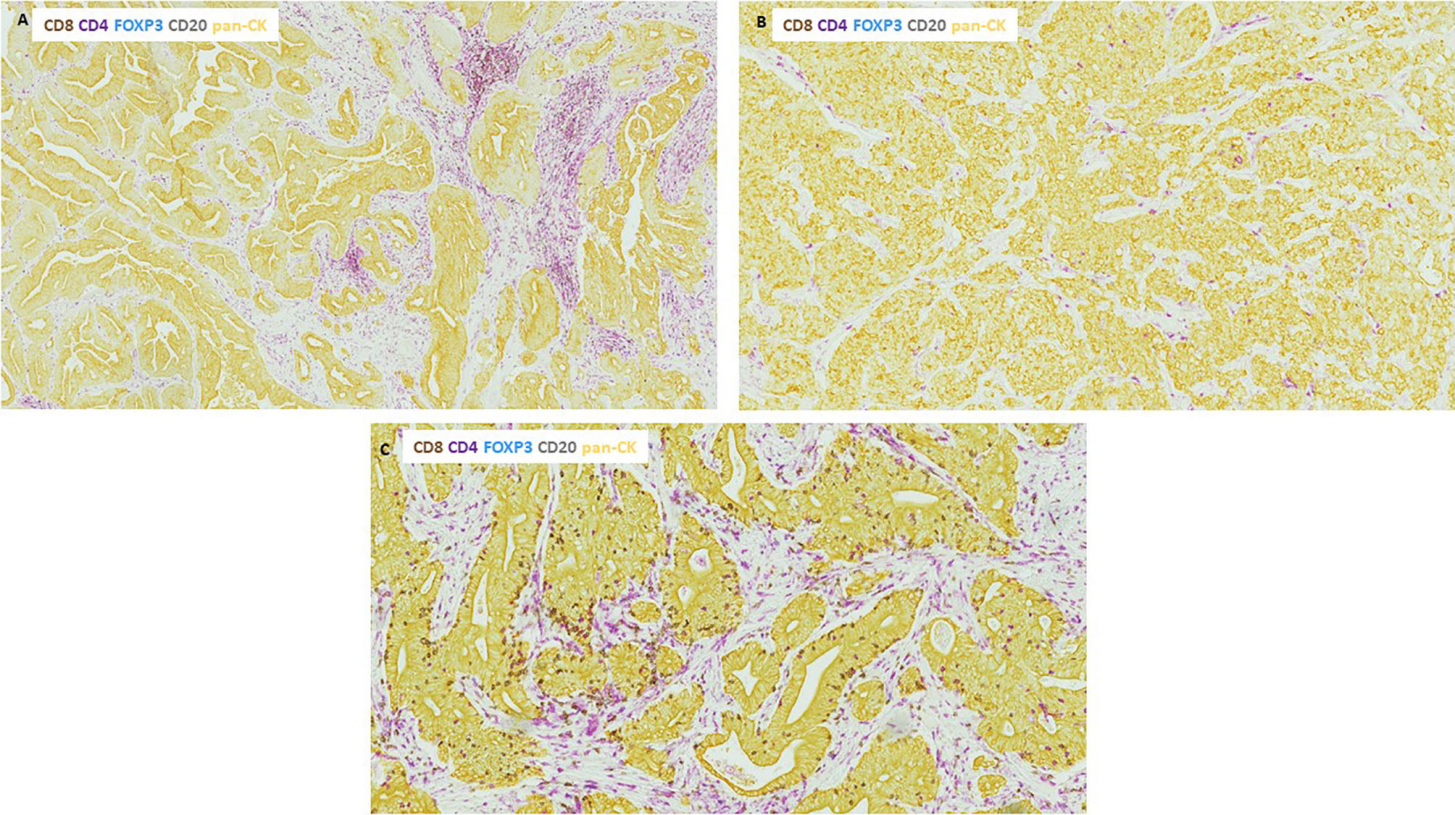
Representative images showing differential immune infiltration in colon cancer tissue. **(A)** Tumor exhibiting patchy immune infiltration consisting of areas with heavy infiltration combined with sparsely infiltrated areas. **(B)** Dense tumor tissue with sparse stroma and modest immune infiltration. **(C)** Highly immune infiltrated tumor with abundant immune cells located within both the tumor tissue and stromal spaces. All images are of the adaptive panel (CD8+ T cells, CD4+ T cells, CD4+foxp3 T cells, CD20+ B cells) of IHC-stained immune markers.

Different patterns of immune infiltration both between and within tumors were present in our cohort. Representative images are shown in **Figure 3**. Some tumors exhibited rich immune infiltration of both the stroma and tumor islets while others had stromal compartments with a more patchy immune infiltrate. Finally, there were tumors with dense tumor tissue, sparse stroma, and modest immune infiltration. There was a trend toward a higher stromal component in systemically inflamed patients, but the tumor–stroma ratio (TSR) did not differ significantly between CRP-high and -low tumors (77 *vs.* 72%, respectively, p = 0.11).

**Figure 4:**
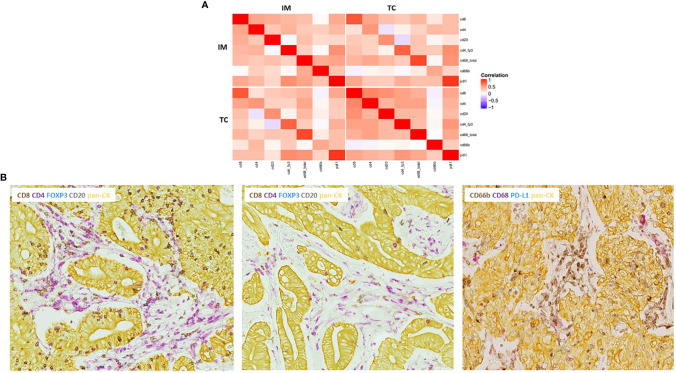
Correlations between immune markers in colon cancer patients. **(A)** Heatmap of Pearson correlation coefficients between individual immune markers at the invasive margin (IM) and tumor center (TC) based upon the combined tumor-infiltrating and stromal immune cell densities. Red color indicates strong positive correlation, blue indicates strong negative correlation, and white indicates no correlation. **(B)** Representative images of existing correlations. Left: tumor slide from the IM of a CRP-low MSS tumor stained with the adaptive IHC panel showing a strong correlation between adaptive immune cells, particularly CD8+ and CD4+ T cells. Middle: tumor slide from the TC of the same patient as in the left panel showing modest adaptive immune infiltration illustrating a low correlation between immune cells at the IM and TC. Right: tumor section stained with the innate IHC panel showing vigorous neutrophil infiltration, with no correlations with any other marker. *Combined tumor-infiltrating and stromal immune cell densities.

With the notable exception of CD66b+ neutrophils, all other immune cells were more prominent at the IM than in the TC with CD68+ macrophages and CD4+ T lymphocytes being the most abundant types of immune cells (**Table S2**). As illustrated in the correlation heat map of tumor-infiltrating immune cells in **Figure 4**, there was a generally low correlation between immune markers at the IM and TC (**Figures 4A, B**). However, several positive correlations existed among adaptive immune cells, particularly in the TC where CD8+ and CD4+ T cells showed a strong positive correlation. Innate immune cells, on the other hand, were less correlated. Most strikingly, neutrophils turned out to be independent of the presence of any other immune marker as no correlations were evident (**Figure 4B**).

### Exploring the Immune Infiltrate in CRP High and Low Colon Cancer Patients According to MSI Status

Based on the finding that MSI status associated with elevated CRP and that no patients in the CRP-low group had MSI-positive tumors, we evaluated the composition of the immune infiltrate in CRP-high and -low patients according to MSI status. As shown in **Table 2**, there were considerable differences in the pattern of immune infiltration between MSS and MSI-high tumors in the systemically inflamed group and MSS tumors in the non-inflamed group, particularly evident in the TC. Specifically, MSI-high tumors were characterized by significantly higher densities of CD8+ T lymphocytes, CD20+ B cells, and tumor-infiltrating CD4+ T cells as well as higher CD66b+ neutrophil and CD68+ macrophage densities and finally upregulation of PD-L1, predominantly expressed on myeloid immune cells (primarily CD68+ macrophages) infiltrating the tumor stroma and to a lesser extent on tumor cells. Interestingly, the density of foxp3 regulatory T cells also differed significantly among the three groups where CRP-low MSS tumors exhibited the highest proportion followed by MSI CRP-high tumors and finally MSS CRP-high tumors. Of note, MSS CRP-high tumors exhibited the lowest lymphoid cell densities and PD-L1 expression but were significantly more myeloid inflamed compared to MSS CRP-low tumors (**Table 2**).

**Table 2:** Adaptive and innate immune markers in CRP-high and -low colon cancer patients according to MSI status.

Immune marker	Index	Area	CRP < 10, MSS	CRP ≥ 30, MSS	CRP ≥ 30, MSI	p value
**CD8+ T cells**	Stroma	TC	0.74 (0.03–4.60)	0.08 (0.00–1.37)	0.86 (0.02–2.56)	**0.049**
	Tumor infiltrating	TC	0.07 (0.00–0.74)	0.02 (0.01–0.14)	0.09 (0.00–0.96)	**0.042**
**CD4+ T cells**	Stroma	TC	1.25 (0.10–3.18)	0.69 (0.21–3.64)	0.72 (0.07–3.84)	0.51
	Tumor infiltrating	TC	0.25 (0.04–1.09)	0.20 (0.04–0.36)	0.65 (0.10–4.63)	**0.055**
CD20+ B cells^*^	Stroma	IM	0.05 (0.00–0.14)	0.05 (0.01–0.33)	0.24 (0.01–0.84)	**0.020**
		TC	0.03 (0.00–0.08)	0.01 (0.00–0.04)	0.04 (0.00–0.24)	**0.046**
**CD4_foxp3+ T cells**	Stroma	TC	0.33 (0.02–1.06)	0.03 (0.00–0.32)	0.09 (0.00–1.54)	**0.009**
	Tumor infiltrating	TC	0.01 (0.00–0.05)	0.00 (0.00–0.03)	0.02 (0.00–0.21)	0.12
**CD68+ macrophages**	Stroma	TC	1.15 (0.56–3.39)	1.81 (0.30–3.85)	1.51 (0.03–3.39)	0.60
		TC	0.47 (0.12–3.31)	0.89 (0.06–2.36)	1.34 (0.00–5.30)	**0.054**
**CD66b+ neutrophils**	Stroma	TC	0.66 (0.03–2.41)	0.59 (0.05–4.05)	1.51 (1.10–20.67)	**0.04**
	Tumor infiltrating	IM	0.05 (0.00–3.54)	0.71 (0.04–3.23)	0.76 (0.05–1.97)	**0.08**
		TC	0.03 (0.00–2.30)	0.86 (0.04–16.72)	1.26 (0.13–45.14)	**<0.001**
**PD-L1+**	Stroma	IM	0.13 (0.01–1.45)	0.02 (0.00–1.84)	0.32 (0.02–0.71)	**0.026**
		TC	0.12 (0.00–0.90)	0.02 (0.00–0.54)	0.09 (0.00–0.52)	**0.021**
	Tumor infiltrating	IM	0.28 (0.00–0.90)	0.03 (0.00–17.11)	0.54 (0.01–15.57)	**0.051**
		TC	0.14 (0.00–4.87)	0.04 (0.00–16.97)	0.63 (0.00–3.63)	0.09
**CD68_PD-L1+ macrophages**	Stroma	TC	0.10 (0.00–1.85)	0.04 (0.00–1.25)	0.28 (0.00–0.88)	0.14
	Tumor infiltrating	IM	0.07 (0.00–1.30)	0.02 (0.00–1.10)	1.09 (0.00–2.27)	**0.031**
		TC	0.02 (0.00–0.60)	0.05 (0.00–1.25)	0.13 (0.00–0.95)	0.074

Immune markers in percent, median (range). p-values were obtained using the Kruskal–Wallis test.

IM, invasive margin; TC, tumor center.

*CD20+ B cells were infiltrating the stroma only.

Bolded values indicate statistical significant p-values.

Analyzing the CRP-high group as a whole, regardless of MSI status, high CD66b+ neutrophils (p=0.04 and 0.001 at the IM and TC, respectively) and high CD68+ macrophages (p=0.04 at the TC) remained significantly associated with elevated CRP in the univariate analysis, as shown in **Table 3**. In contrast, the adaptive immune markers CD8+ T lymphocytes (p = 0.03 at IM) and foxp3 regulatory T cells (p = 0.03 at TC) correlated inversely with high CRP.

**Table 3:** Associations between selected immune markers and systemic inflammation in colon cancer patients.

Immune marker	Area	Risk of high CRP		p value
		Low, N, % (CI)	High, N, % (CI)	
CD8+ T cells^*^	IM	8, 50 (25–75) %	11, 69 (41–89) %	0.47
** **	TC	13, 81 (54–96) %	6, 38 (15–65) %	**0.03**
CD4+ T cells^**^	IM	9, 56 (30–80) %	10, 63 (35–85) %	1.00
** **	TC	11, 69 (41–89) %	8, 50 (25–75) %	0.47
CD20+ B cells^***^	IM	8, 50 (25–75) %	11, 69 (41–89) %	0.47
** **	TC	11, 69 (41–89) %	8, 50 (25–75) %	0.47
CD4_foxp3+ T cells^***^	IM	11, 69 (41–89) %	8, 50 (25–75) %	0.47
** **	TC	13, 81 (54–96) %	6, 38 (15–65) %	**0.03**
CD68+ macrophages^**^	IM	8, 44 (22–69) %	13, 72 (47–90) %	0.18
** **	TC	7, 39 (17–64) %	14, 78 (52–94) %	**0.04**
CD66b+ neutrophils^**^	IM	7, 39 (17–64) %	14, 78 (52–94) %	**0.04**
** **	TC	5, 28 (10–53) %	16, 89 (65–99) %	**<0.001**
PD-L1+^**^	IM	12, 67 (41–87) %	9, 50 (26–74) %	0.50
** **	TC	10, 56 (31–78) %	11, 61 (36–83) %	1.00
CD68_PD-L1+ macrophages^*^	IM	9, 50 (26–74) %	12, 67 (41–87) %	0.50
	TC	10, 56 (31–78) %	11, 61 (36–83) %	1.00

Number, risk (CI) of CPR ≥ 30. Univariate analysis.

High and low are categorized as above or below the median of individual immune markers.

*Composite score of immune cells in the stroma and directly tumor infiltrating.

**Tumor infiltrating only.

***Stroma only.

IM, invasive margin; TC, tumor center.

Bolded values indicate statistical significant p-values.

Despite the relatively low number of events in our cohort, survival analyses were performed, as shown in **Table S3**. Of particular interest, CD68+ macrophages at the IM correlated with risk of death from colon cancer (39% (CI: 17–64) for high CD68+ versus 0% (CI: 0–19) for low CD68+, p = 0.008) whereas stromal CD20+ B cells at the TC correlated with risk of death from all causes (50% (CI: 25–75) for low CD20+ versus 13% (CI: 2–38) for high CD20+, p = 0.05). Neither neutrophils nor CD8+ T cells had prognostic impact in our cohort. Due to the small number of patients and few events, multivariate analyses were not performed on this material.

### Systemic Inflammation Associates With a Myeloid Inflamed Tumor Microenvironment in CC Patients

**Figure 5:**
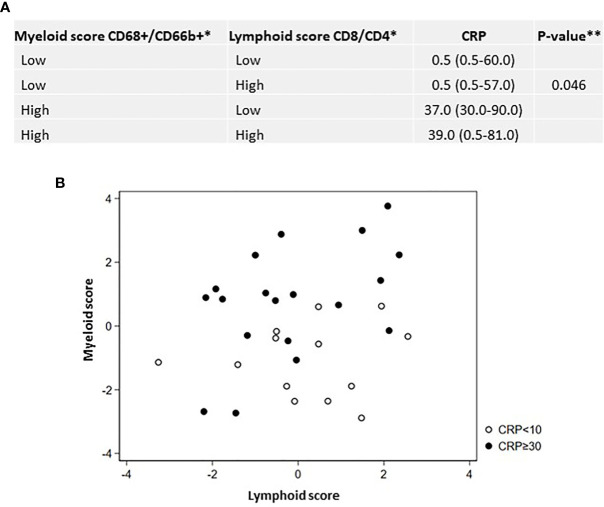
Composite lymphoid and myeloid immune scores correlate differentially with systemic inflammation. **(A)** Median (range) CRP by myeloid *vs.* lymphoid composite immune scores. **(B)** Lymphoid and myeloid composite scores in CRP-high and -low patients. *Immune scores based upon directly tumor-infiltrating immune cell densities at the tumor center. Lymphoid composite score: compounded densities of CD8+T lymphocytes and CD4+T lymphocytes. Myeloid composite score: compounded densities of CD68+ macrophages (inclusive CD68PDL1+) and CD66b+ neutrophiles. **p-value obtained using the Kruskal–Wallis test comparing all four groups.

We hypothesized that a combinatorial approach based on the expression of two or more immune markers rather than single-cell analysis better could elucidate potential correlations between distinct immune phenotypes and systemic inflammation. For that purpose, densities of CD4+ and CD8+ T- lymphocytes (termed the adaptive composite score) and CD68+ macrophages and CD66b+ neutrophils (termed the innate composite scores) were compounded and categorized as high or low based on the median of the combined scores. Interestingly, we found that regardless of the adaptive score, tumors with a high innate score had increased risk of elevated CRP (shown in **Figure 5A**). The scatter plot in **Figure 5B** depicting adaptive and innate composite scores in CRP-high and -low patients further supported this observation, suggesting that it is the presence of a myeloid-inflamed and not the absence of a lymphoid-inflamed TME that seems to be the driver of systemic inflammation. 

### Different Immune Phenotypes Correlate With MSI Status and Systemic Inflammation

**Figure 6:**
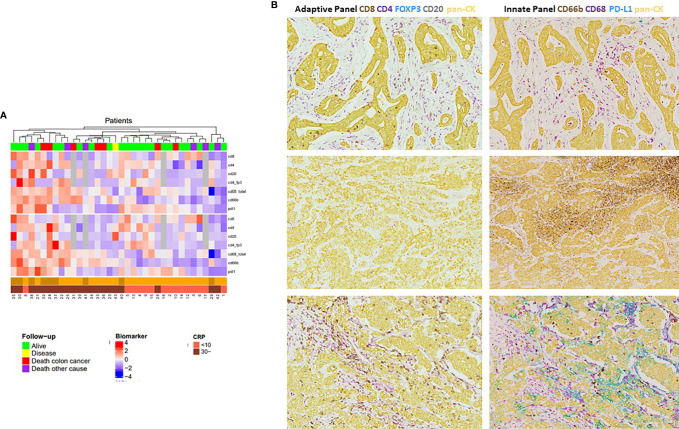
Hierarchical cluster analysis of selected adaptive and innate immune markers in primary resected colon cancer patients. Heat map of unsupervised hierarchical clustering based upon the densities of tumor-infiltrating immune markers. Data were log-transformed to get a normal distribution, standardized to mean zero and variance 1. Information on CRP values; MSI and follow-up statuses were added to the dendrogram for visual interpretation after performing the cluster analysis. **(A)** Clustering based upon tumor-infiltrating immune cell densities and PD-L1 tumor expression. Red color indicates high density, blue low density of each immune marker. **(B)** Representative images from patients with the three predominant immune phenotypes present within our cohort. Upper panel: mIHC-stained tumor slides from a CRP low, MSS pt. with prominent lymphoid infiltration and modest myeloid immune infiltration. Middle panel: tumor slides from a CRP-high, MSS pt. showing predominant myeloid infiltration (almost exclusively CD66b+ neutrophils) and only marginal infiltration by lymphoid immune cells (CD4+ T cells only). Lower panel: tumor slides from a CRP-high, MSI-positive pt. being hyperinflamed with vigorous lymphoid and myeloid immune infiltration and high PD-L1 expression, predominantly expressed by CD68+ macrophages.

To further explore the concept of differential immune phenotypes, present in our cohort, hierarchical clustering was performed identifying subgroups of tumors with distinct immunological features. As shown in **Figure 6**, three clusters seemed to be present consisting of a subgroup of tumors predominantly lymphoid-inflamed, a subgroup that was more myeloid-inflamed, and a group of hyper-inflamed tumors with high densities of both lymphoid and myeloid immune cells. Additionally, we identified a small group of hypo-inflamed tumors with low numbers of both types of tumor-infiltrating immune cells. When adding information on CRP values and MSI status in the heat map, systemically inflamed MSI-positive tumors corresponded quite well with the group of hyper-inflamed tumors, whereas MSS CRP-high tumors corresponded with the ones being more myeloid and less lymphoid inflamed and, finally, MSS CRP-low tumors seemed either predominantly lymphoid or hypo-inflamed. Of note, none of the CRP-low tumors exhibited high scores of myeloid immune cells.

### Spatial Distribution of Tumor Infiltrating Neutrophils Correlates With Systemic Inflammation

**Figure 7:**
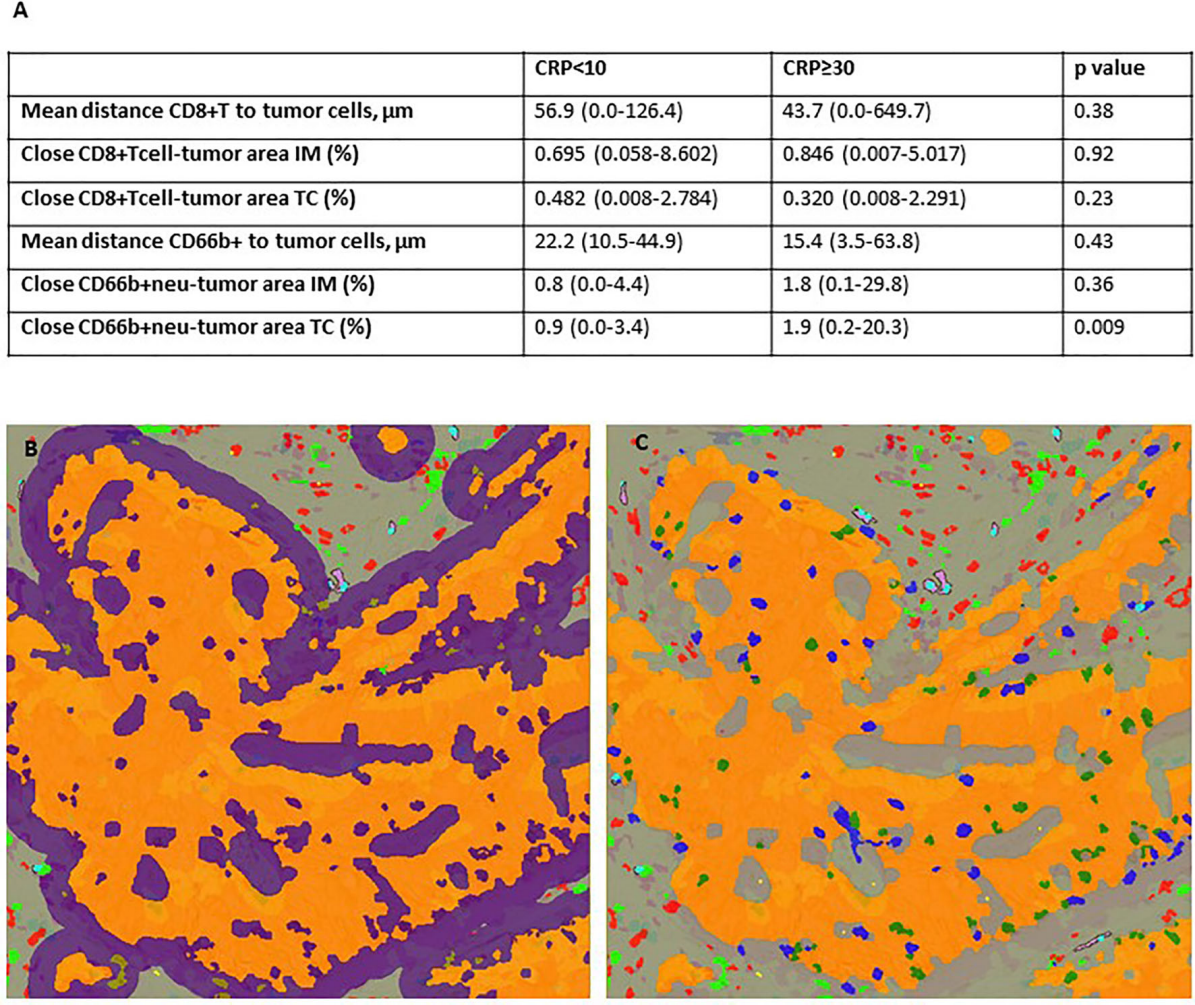
Spatial distribution of CD8+T-cells and neutrophils in CRP-high and -low colon cancer patients. **(A)** Table showing median (range) of various spatial relationships. p-values were obtained using the Kruskal–Wallis test. **(B)** Close immune cells to the tumor area of either CD8+ T cells or CD66b+ neutrophils estimated by outlining 20 μm around tumor islet. **(C)** Nearest neighbor analysis estimating the average distance between immune cells of interest (either CD8+ T cells or CD66b+ neutrophils) and nearest tumor cells.

Given the assumption that the combined information on both the precise localization and density of immune cells reflects cell functionality and potential interactions taking place within the TME, we investigated the spatial distribution of CD8+ lymphocytes and CD66b+ neutrophils in CRP-high and -low patients. As shown in **Figure 7**, systemically inflamed tumors exhibited significantly higher density of neutrophils in close proximity to tumor nests compared with non-inflamed tumors (1.9% *vs.* 0.9%, respectively, p = 0.009). Moreover, there was a tendency toward lower mean distance between neutrophils and tumor cells in the systemically inflamed patients. We found no significant differences in the spatial distribution of CD8+ lymphocytes between CRP-high and -low tumors. Based on the proof-of-concept approach of the study, further spatial analyses were not performed on this material.

## Discussion

In this study, we explored the tumor-immune microenvironment in colon cancer patients related to the presence of SIR, covering important players of both adaptive and innate immunity and their spatial distribution within the primary tumors. By analyzing the immune contexture in patients with and without accompanying SIR, we revealed upregulation of myeloid features in the TME from systemically inflamed patients. Specifically, and in line with our hypothesis, tumor-infiltrating neutrophils and macrophages associated with systemic inflammation. Most strikingly, we found that regardless of an adaptive composite score (compounded CD4+ and CD8+ T cells), a high innate score (compounded CD66b+ neutrophils and CD68+ macrophages) significantly increased the risk of elevated CRP, indicating that it is the presence of a myeloid-inflamed and not the absence of a lymphoid-inflamed TME that associates with systemic inflammation.

A strong impact of myeloid cells has also been demonstrated in a squamous head and neck cancer cohort revealing differential immune profiles, representing either lymphoid-, myeloid-, or hypo-inflamed tumors, where myeloid-enriched tumors associated with the shortest overall survival regardless of HPV status (19). Additionally, in a small validation cohort of pancreatic cancer patients receiving a neoadjuvant CSF vaccine, tumors seemed to cluster into two groups depending on the degree of myeloid inflammation, where again myeloid-dominated tumors correlated with the poorest clinical outcomes (19). Notably, the lymphocyte infiltration did not differ between the two groups, indicating a strong immunosuppressive role of myeloid cells potentially compromising effective antitumor immune responses. A detrimental effect of myeloid cells was also found in a recent study using a transgenic mouse model of HPV-derived cancers treated with a therapeutic vaccine alone or in combination with double immune checkpoint blockade (20). In this study, vaccination alone or in combination elicited neither tumor regression nor effective CD8+ responses due to the expansion of myeloid cells in peripheral lymphoid tissue, suggesting a systemic myeloid-driven immunosuppression impairing the efficacy of immunotherapy.

These results combined with the findings of our study highlight the strong role myeloid cells may play in the TME by creating an immunosuppressive state and even outperform the potential beneficial role of lymphoid cells and negatively affect prognosis. Although myeloid cells have been associated with poor survival and treatment outcome in several cancer types (21–24), their role in the TME remains to be fully understood and undervalued compared with the much more studied lymphoid cells (11, 14). Experimental studies have shown that both macrophages and neutrophils, being some of the most important players of innate immunity, may exhibit contradictory roles in cancer with both pro-tumoral and antitumoral properties depending on the immunological context (13, 25, 26). Major tumor-promoting and immunosuppressive functions of myeloid cells include release of growth factors such as MMP-9 and oncostatin M which induce upregulation of VEGF and HIF-2alpha pathways leading to neo-angiogenesis, hypoxia, and ultimately cancer invasiveness and progression (14, 27, 28). Moreover, myeloid cells have been shown to be crucial at all steps of the metastatic process (26, 29, 30). In addition to their direct tumor-promoting functions, both tumor-associated macrophages (TAMs) and tumor-associated neutrophils (TANs) may also suppress antitumor adaptive immune responses through the production of IL-10 and TGF-beta as well as the enzymes arginase 1 and IDO, which are detrimental for T cell-mediated immunity (13, 31).

It is particularly interesting to see what happens under circumstances of chronic wounding, which might be analogous to the situation of the colon where tumors can arise in relation to a chronically inflamed and often injured epithelium (32). Using a zebrafish melanoma model, it has been shown that neutrophils attracted to a wound are rapidly diverted to adjacent pre-neoplastic cells resulting in increased proliferation and melanoma formation (33).The corresponding clinical evidence for such a direct neutrophil-driven tumor growth has been further demonstrated in human melanoma where neutrophil density correlated strongly with increased proliferation and associated with poor melanoma-specific survival (33).

Altogether, these exciting findings support the notion that neutrophils may fuel and shape tumors and highlight innate immune cells as a therapeutic target for immunotherapeutic approaches (34).

By far, local and systemic tumor-associated inflammations have been regarded as separate processes with only few studies investigating their possible interrelationship (35–38). Similar to our findings, a recent study in all stages of CRC demonstrated a significant inverse relationship between high CRP (>10 mg/l) and foxp3 regulatory T cells, but no associations were detected for other immune cells including myeloid cell types. Of note and contrary to our findings, no significant relationship was found between MSI status and CRP except that all patients with CRP>75 mg/l had MSS tumors (39). However, the immune infiltration in this study was determined using TMAs. Based on the intratumoral heterogeneity observed in our material, it could be that the tissue sampling performed when preparing TMAs, being snapshots of the tumor, is not representative of the global immune cell infiltration and may in part explain the lack of association. Additionally, this study also included rectal cancer patients which have been shown to be less systemically inflamed and might represent another tumor entity when it comes to the inflammatory tumor reaction (40).

An intriguing and initially surprising finding of our study was the significant association between positive MSI status and systemic inflammation. Given the good prognosis related to MSI in early-stage colon cancer and the poor prognosis related to the SIR, one might rather expect an inverse or no association between the two entities. Nevertheless, we found that MSI CRP-high tumors not only were hyper-inflamed in terms of considerable lymphoid inflammation that previously has been shown to accompany MSI-positive tumors, but also were highly infiltrated by myeloid immune cells, particularly neutrophils. Moreover, MSI-high tumors exhibited upregulation of PD-L1, predominantly expressed by myeloid immune cells infiltrating the tumor stroma and to a lesser extent by the tumor cells themselves. This observation stands in contrast to other tumor types such as lung, bladder, and kidney cancer, where tumor PD-L1 expression is a common feature (41). However, consistent with our findings, a study by Llosa et al. demonstrated much higher levels of PD-L1 expression in MSI compared to MSS tumors, almost exclusively expressed by tumor-infiltrating myeloid cells and not the tumor cells (42). Indeed, our findings need to be further explored in a larger dataset, but a working hypothesis could be that MSI tumors accompanied by systemic inflammation exhibit a highly myeloid immune infiltrated TME resulting in an immunosuppressive state either caused by 1) a compensatory upregulation of immune checkpoints stimulated by preexisting cytokines such as IFN-gamma following the MSI-induced active immune microenvironment leading to a functional exhaustion of the T cells or 2) direct immunosuppressive and tumor-promoting effects exerted by the myeloid cells themselves. In either way or both, such myeloid-dependent immunosuppression might counterbalance the potential beneficial effects of the lymphoid immune infiltration and blunt effective antitumor immune responses, at least without immune checkpoint inhibition.

In an effort to decipher the complex TME and variable treatment outcomes to immunotherapy, emerging studies take into context the spatial aspect of the tumor immune landscape (16, 43, 44). Recent data point toward both prognostic and predictive values of proximity analyses, in terms of measurement of the exact localization and distances between tumor and immune cells, suggesting that spatial patterns reflect cell functionality and clinically meaningful tumor–host interactions taking place within the TME (45–47). Notably, in our study we found that systemically inflamed patients had significantly more neutrophils in close proximity to tumor cells as compared to non-inflamed patients whereas no differences in the spatial features of CD8+ T cells could be detected. Again, this finding supports the hypothesis that myeloid inflammation and neutrophils in particular play a critical role in the context of SIR in CC. Additionally, it adds to the argumentation for preferring whole slides over TMAs enabling a more comprehensive mapping of the immune context of tumors (48).

Our study has several limitations. Due to the proof-of concept design, it covers a limited patient series. Thus, our findings need to be tested in a larger dataset before biologic conclusions can be drawn. We plan to enrich the cohort for confirmation and further analyses to expand our understanding of how systemic inflammation and localized tumor-associated inflammation influence each other. Another limitation owing to the IHC itself is the challenge of characterizing functional phenotypes. Myeloid cells exhibit a high degree of plasticity displaying a continuum of polarization states being more or less immuno-suppressive or stimulatory. This dynamic diversity is difficult to capture with IHC antibodies directed toward one or two fixed cell markers (28). Although we performed spatial analyses as a pseudo marker of cell functionality, the precise identification of the multitude of polarization states that seem to exist for myeloid cells cannot be truly captured by current IHC techniques (49).

Taken together, our data highlight the importance of a broader and more comprehensive immune characterization of tumors covering both lymphoid and myeloid cell populations. The concept of hot and cold tumors, categorizing tumors based on the infiltration of T cells, has been widely used to inform patient prognosis and predict immunotherapeutic efficacy (50). Within recent years, this simplistic classification has been refined acknowledging the complexity and heterogeneity of the immune infiltrate of tumors with the introduction of four distinct immune subgroups: hot, altered-excluded, altered-immunosuppressed, and cold (51). However, this approach is still mainly focusing on T-cell infiltration without further characterizing other cell populations such as myeloid immune cells. Our findings, supported by others, demonstrate the potential limitations of such a T cell-focused classification, indicating that “hot tumors” can be so much more than just “T cell inflamed.” We hypothesize that a vigorous myeloid-inflamed TME might counterbalance the beneficial and potential tumor-suppressive effect of a strong lymphoid immune infiltrate and negatively affect antitumor immunity. Accordingly, we propose that strategies of converting “cold tumors to hot” also should include efforts of targeting the myeloid-derived immunosuppression before harnessing T cell-mediated antitumor immune responses.

In conclusion, we herein provide a framework for expanding our understanding of the immune landscape in CC and explore the role of CRP as a systemic and informative biomarker of the immune responses taking place at the tumor site. Further deciphering distinct immune phenotypes and spatial features that associate with systemic inflammation may improve our understanding of inherent immune responses in CC and hold critical implications for therapeutic approaches.

## Data Availability Statement

The original contributions presented in the study are included in the article/**Supplementary Material**. Further inquiries can be directed to the corresponding author. 

## Ethics Statement

The studies involving human participants were reviewed and approved by the Regional Committees for Medical and Health Research Ethics (REC) South East. Written informed consent for participation was not required for this study in accordance with the national legislation and the institutional requirements. 

## Author Contributions

AK, the first author, has performed lab work, planned and performed digital analyses, participated in statistics together with EP, written the draft for the manuscript, and driven the scientific discussions. PN assisted in performing, optimizing, and interpreting digital imaging. JG planned and performed laboratory work. MN reviewed and annotated tumor regions from FFPE tissue blocks and evaluated the MMR IHC-analysis. TS and CK participated in methodological and scientific discussions and had the main ideas behind the study. All authors have reviewed and commented the manuscript during the process and before submission. All authors contributed to the article and approved the submitted version. 

## Conflict of Interest

The authors declare that the research was conducted in the absence of any commercial or financial relationships that could be construed as a potential conflict of interest.

## Publisher’s Note

All claims expressed in this article are solely those of the authors and do not necessarily represent those of their affiliated organizations, or those of the publisher, the editors and the reviewers. Any product that may be evaluated in this article, or claim that may be made by its manufacturer, is not guaranteed or endorsed by the publisher.
