# Supplementary material for: Systemic Inflammation Associates With a Myeloid Inflamed Tumor Microenvironment in Primary Resected Colon Cancer—May Cold Tumors Simply Be Too Hot?
Source: Front Immunol. 2021 Aug 31;12:716342. doi: 10.3389/fimmu.2021.716342 (PMC8438238; doi:10.3389/fimmu.2021.716342)
Supplement: Supplementary file 1 [file DataSheet_1.docx]

**Supplementary Table 1 Primary antibodies with corresponding incubation times used for chromogenic multiplex IHC**

| **Adaptive Immune panel** | | |  |  |  |
| --- | --- | --- | --- | --- | --- |
| **Target** | **Clone** | **Vendor** | **Specie** | **Dilution** | **Incubation** |
| CD8 | C8/144B | Dako | Mouse | 1:150 | 40min |
| CD4 | sp35 | Ventana | rabbit | RTU | 48min |
| fox-p3 | sp97 | Abcam | rabbit | 1:10 | 60min |
| pan-CK | Ae1/3 | Dako | Mouse | 1:100 | 32min |
| CD20 | L26 | Ventana | Mouse | RTU | 32 min |
| **Innate Immune panel** | | |  |  |  |
| CD66b | G10F5 | Beckman Coulter | mouse | 1:200 | 40min |
| CD68 | PG-M1 | Dako | mouse | 1:100 | 32min |
| PD-L1 | sp263 | Ventana | rabbit | RTU | 60min |
| pan-CK | Ae1/3 | Dako | mouse | 1:100 | 32min |

**Supplementary Table 2 Adaptive and Innate immune markers in CRP-high and low colon cancer patients**

| **Immune Marker** | **Index** | **Area** | **CRP<10** | **CRP≥30** | **P-value** |
| --- | --- | --- | --- | --- | --- |
| **CD8+ T cells** | All ROI | IM | 0.95 (0.10-13.56) | 1.15 (0.01-3.92) | 0.89 |
|  |  | TC | 0.59 (0.03-3.29) | 0.22 (0.01-1.78) | 0.09 |
| **CD4+ T cells** | All ROI | IM | **1.55 (0.07-3.83)** | **1.65 (0.29-6.50)** | 0.35 |
|  |  | TC | **0.95 (0.09-2.40)** | **0.58 (0.07-2.90)** | 0.31 |
| **CD20+ B cells** | Stroma | IM | 0.05 (0.00-0.14) | 0.10 (0.01-0.84) | 0.053 |
|  |  | TC | 0.03 (0.00-0.08) | 0.02 (0.00-0.24) | 0.27 |
| **CD4_fp3 T cells** | All ROI | IM | 0.21 (0.04-1.64) | 0.19 (0.00-0.63) | 0.13 |
|  |  | TC | 0.18 (0.02-0.59) | 0.04 (0.00-1.10) | 0.003 |
| **CD68+ macrophages** | All ROI | IM | **1.69 (0.47-3.77)** | **3.04 (0.00-7.93)** | 0.056 |
|  |  | TC | **0.92 (0.43-3.40)** | **1.48 (0.03-3.58)** | 0.11 |
| **CD66b+ neutrophils** | All ROI | IM | 0.39 (0.00-2.70) | 1.46 (0.06-21.09) | 0.09 |
|  |  | TC | 0.50 (0.03-2.39) | 1.06 (0.11-28.86) | 0.002 |

Cell densities in percent of total area of the tumor compartment, median (range)

ROI: Region of interest, composite score of immune cells in the stroma and directly tumor infiltrating

IM: Invasive Margin TC: Tumor Center

**Supplementary Table 3: Associations between selected immune markers and death from colon cancer or other causes in colon cancer patients**

| **IM** | | | |  |
| --- | --- | --- | --- | --- |
| **Biomarker** | **N** | **Outcome** | **Event, risk % (CI)** | **P-value** |
| **CD8+ T cells (Stroma)** | | | | |
| Low | 16 | Death of all causes | 4, 25 (7-52) | 0.70 |
| High | 16 |  | 6, 38 (15-65) |  |
| **CD8+ T cells (Stroma)** | | | | |
| Low | 16 | Death of colon cancer | 2, 13 (2-38) | 0.65 |
| High | 16 |  | 4, 25 (7-52) |  |
| **CD8+ T cells (Tumor)** | | | | |
| Low | 16 | Death of all causes | 5, 31 (11-59) | 1.00 |
| High | 16 |  | 5, 31 (11-59) |  |
| **CD8+ T cells (Tumor)** | | | | |
| Low | 16 | Death of colon cancer | 3, 19 (4-46) | 1.00 |
| High | 16 |  | 3, 19 (4-46) |  |
| **CD4+ T cells (Stroma)** | | | | |
| Low | 16 | Death of all causes | 6, 38 (15-65) | 0.70 |
| High | 16 |  | 4, 25 (7-52) |  |
| **CD4+ T cells (Stroma)** | | | | |
| Low | 16 | Death of colon cancer | 2, 13 (2-38) | 0.65 |
| High | 16 |  | 4, 25 (7-52) |  |
| **CD20+ B cells (Stroma)** | | | | |
| Low | 16 | Death of all causes | 5, 31 (11-59) | 1.00 |
| High | 16 |  | 5, 31 (11-59) |  |
| **CD20+ B cells (Stroma)** | | | | |
| Low | 16 | Death of colon cancer | 3, 19 (4-46) | 1.00 |
| High | 16 |  | 3, 19 (4-46) |  |
| **CD4_foxp3+ T cells (Stroma)** | | | | |
| Low | 16 | Death of all causes | 5, 31 (11-59) | 1.00 |
| High | 16 |  | 5, 31 (11-59) |  |
| **CD4_foxp3+ T cells (Stroma)** | | | | |
| Low | 16 | Death of colon cancer | 2, 13 (2-38) | 0.65 |
| High | 16 |  | 4, 25 (7-52) |  |
| **CD68+ macrophages (Stroma)** | | | | |
| Low | 18 | Death of all causes | 4, 22 (6-48) | 0.16 |
| High | 18 |  | 9, 50 (26-74) |  |
| **CD68+ macrophages(Stroma)** | | | | |
| Low | 18 | Death of colon cancer | 0, 0 (0-19) | 0.008 |
| High | 18 |  | 7, 39 (17-64) |  |
| **CD68+ macrophages (Tumor)** | | | | |
| Low | 18 | Death of all causes | 4, 22 (6-48) | 0.16 |
| High | 18 |  | 9, 50 (26-74) |  |
| **CD68+ macrophages (Tumor)** | | | | |
| Low | 18 | Death of colon cancer | 1, 6 (0-27) | 0.09 |
| High | 18 |  | 6, 33 (13-59) |  |
| **CD66b+ neutrophils (Stroma)** | | | | |
| Low | 18 | Death of all causes | 6, 33 (13-59) | 1.00 |
| High | 18 |  | 7, 39 (17-64) |  |
| **CD66b+ neutrophils (Stroma)** | | | | |
| Low | 18 | Death of colon cancer | 3, 17 (4-41) | 1.00 |
| High | 18 |  | 4, 22 (6-48) |  |
| **CD66b+ neutrophils (Tumor)** | | | | |
| Low | 18 | Death of all causes | 6, 33 (13-59) | 1.00 |
| High | 18 |  | 7, 39 (17-64) |  |
| **CD66b+ neutrophils (Tumor)** | | | | |
| Low | 18 | Death of colon cancer | 2, 11 (1-35) | 0.40 |
| High | 18 |  | 5, 28 (10-53) |  |
| **PDL1+ (Tumor)** | | | | |
| Low | 18 | Death of all causes | 8, 44 (22-69) | 0.49 |
| High | 18 |  | 5, 28 (10-53) |  |
| **PDL1 (Tumor)** | | | | |
| Low | 18 | Death of colon cancer | 3, 17 (4-41) | 1.00 |
| High | 18 |  | 4, 22 (6-48) |  |
| **CD68_PDL1+ (Stroma)** | | | | |
| Low | 18 | Death of all causes | 5, 28 (10-53) | 0.49 |
| High | 18 |  | 8, 44 (22-69) |  |
| **CD68_PDL1+ (Stroma)** | | | | |
| Low | 18 | Death of colon cancer | 2, 11 (1-35) | 0.40 |
| High | 18 |  | 5, 28 (10-53) |  |
| **TC** | | | | |
| **Biomarker** | **N** | **Outcome** | **Event, risk % (CI)** | **P-value** |
| **CD8+ T cells (Stroma)** | | | | |
| Low | 16 | Death of all causes | 6, 38 (15-65) | 0.70 |
| High | 16 |  | 4, 25 (7-52) |  |
| **CD8+ T cells (Stroma)** | | | | |
| Low | 16 | Death of colon cancer | 3, 19 (4-46) | 1.00 |
| High | 16 |  | 3, 19 (4-46) |  |
| **CD8+ T cells (Tumor)** | | | | |
| Low | 16 | Death of all causes | 6, 38 (15-65) | 0.70 |
| High | 16 |  | 4, 25 (7-52) |  |
| **CD8+ T cells (Tumor)** | | | | |
| Low | 16 | Death of colon cancer | 4, 25 (7-52) | 0.65 |
| High | 16 |  | 2, 13 (2-38) |  |
| **CD4+ T cells (Stroma)** | | | | |
| Low | 16 | Death of all causes | 5, 31 (11-59) | 1.00 |
| High | 16 |  | 5, 31 (11-59) |  |
| **CD4+ T cells (Stroma)** | | | | |
| Low | 16 | Death of colon cancer | 1, 6 (0-30) | 0.17 |
| High | 16 |  | 5, 31 (11-59) |  |
| **CD20+ B cells (Stroma)** | | | | |
| Low | 16 | Death of all causes | 8, 50 (25-75) | 0.05 |
| High | 16 |  | 2, 13 (2-38) |  |
| **CD20+ B cells (Stroma)** | | | | |
| Low | 16 | Death of colon cancer | 5, 31 (11-59) | 0.17 |
| High | 16 |  | 1, 6 (0-30) |  |
| **CD4_foxp3+ T cells (Stroma)** | | | | |
| Low | 16 | Death of all causes | 5, 31 (11-59) | 1.00 |
| High | 16 |  | 5, 31 (11-59) |  |
| **CD4_foxp3+ T cells (Stroma)** | | | | |
| Low | 16 | Death of colon cancer | 2, 13 (2-38) | 0.65 |
| High | 16 |  | 4, 25 (7-52) |  |
| **CD68+ macrophages (Stroma)** | | | | |
| Low | 18 | Death of all causes | 4, 22 (6-48) | 0.16 |
| High | 18 |  | 9, 50 (26-74) |  |
| **CD68+ macrophages (Stroma)** | | | | |
| Low | 18 | Death of colon cancer | 2, 11 (1-35) | 0.40 |
| High | 18 |  | 5, 28 (10-53) |  |
| **CD68+ macrophages (Tumor infiltrated)** | | | | |
| Low | 18 | Death of all causes | 6, 33 (13-59) | 1.00 |
| High | 18 |  | 7, 39 (17-64) |  |
| **CD68+ macrophages (Tumor infiltrated)** | | | | |
| Low | 18 | Death of colon cancer | 4, 22 (6-48) | 1.00 |
| High | 18 |  | 3, 17 (4-41) |  |
| **CD66b+ neutrophils (Stroma)** | | | | |
| Low | 18 | Death of all causes | 6, 33 (13-59) | 1.00 |
| High | 18 |  | 7, 39 (17-64) |  |
| **CD66b+ neutrophils (Stroma)** | | | | |
| Low | 18 | Death of colon cancer | 4, 22 (6-48) | 1.00 |
| High | 18 |  | 3, 17 (4-41) |  |
| **CD66b+ neutrophils (Tumor)** | | | | |
| Low | 18 | Death of all causes | 7, 39 (17-64) | 1.00 |
| High | 18 |  | 6, 33 (13-59) |  |
| **CD66b+ neutrophils (Tumor)** | | | | |
| Low | 18 | Death of colon cancer | 3, 17 (4-41) | 1.00 |
| High | 18 |  | 4, 22 (6-48) |  |
| **PDL1+ (Tumor)** | | | | |
| Low | 18 | Death of all causes | 6, 33 (13-59) | 1.00 |
| High | 18 |  | 7, 39 (17-64) |  |
| **PDL1+ (Tumor)** | | | | |
| Low | 18 | Death of colon cancer | 2, 11 (1-35) | 0.40 |
| High | 18 |  | 5, 28 (10-53) |  |
| **CD68_PDL1+ (Stroma)** | | | | |
| Low | 18 | Death of all causes | 7, 39 (17-64) | 1.00 |
| High | 18 |  | 6, 33 (13-59) |  |
| **CD68_PDL1+ (Stroma)** | | | | |
| Low | 18 | Death of colon cancer | 3, 17 (4-41) | 1.00 |
| High | 18 |  | 4, 22 (6-48) |  |

Univariate analysis. Immune markers categorized as high or low according to the median value. IM=Invasive Margin, TC=Tumor Center
